# Engaging Older Veterans With Serious Mental Illness in Physical Activity: In-Person, Remote, and Hybrid Models

**DOI:** 10.1093/geroni/igab046.787

**Published:** 2021-12-17

**Authors:** Sera Havrilla, Alicia Lucksted, Deborah Medoff, Karen Fortuna, Amanda Peeples, Anjana Muralidharan

**Affiliations:** 1 VISN 5 MIRECC, Baltimore, Maryland, United States; 2 University of Maryland School of Medicine, Baltimore, Maryland, United States; 3 Dartmouth College, Lebanon, New Hampshire, United States; 4 Veterans Affairs Capitol Healthcare Network, Baltimore, Maryland, United States

## Abstract

Older adults with serious mental illness (SMI) have complex care needs across medical, psychiatric, cognitive, and social domains. This growing population exhibits high levels of medical comorbidity and sedentariness. Innovative interventions that promote holistic recovery for this group are needed, especially in the context of the COVID-19 pandemic. Peer Education on Exercise for Recovery (PEER) is a peer coaching intervention, delivered by VA Peer Specialists (Veterans with lived experience of mental illness), to promote exercise and physical activity among older adults with SMI. This paper will present on three different models of PEER: fully in-person, fully remote, and a hybrid model with both in-person and remote elements. Preliminary data indicates that PEER is (1) engaging and well-liked, (2) associated with greater sustained increases in physical activity compared to an active control, and (3) can lead to sustained physical activity increases that are resilient to situational constraints such as physical distancing.

